# Effect of Anti-Inflammatory Diets on Pain in Rheumatoid Arthritis: A Systematic Review and Meta-Analysis

**DOI:** 10.3390/nu13124221

**Published:** 2021-11-24

**Authors:** Katja A. Schönenberger, Anne-Catherine Schüpfer, Viktoria L. Gloy, Paul Hasler, Zeno Stanga, Nina Kaegi-Braun, Emilie Reber

**Affiliations:** 1 Department of Diabetes, Endocrinology, Nutritional Medicine and Metabolism, Inselspital, Bern University Hospital, University of Bern, 3010 Bern, Switzerland; anne-catherine.schuepfer@students.unibe.ch (A.-C.S.); zeno.stanga@insel.ch (Z.S.); emilie.reber@insel.ch (E.R.); 2 Division of Clinical Pharmacy and Epidemiology, Department of Pharmaceutical Sciences, University of Basel, 4031 Basel, Switzerland; 3 Basel Institute for Clinical Epidemiology and Biostatistics, Department of Clinical Research, University Hospital Basel, University of Basel, 4031 Basel, Switzerland; viktoria.gloy@usb.ch; 4 Division of Rheumatology, Medical University Department, University of Basel Medical Faculty, Kantonsspital Aarau, 5001 Aarau, Switzerland; paul.hasler@ksa.ch; 5 Division of General Internal and Emergency Medicine, Medical University Department, University of Basel Medical Faculty, Kantonsspital Aarau, 5001 Aarau, Switzerland; nina.kaegi@ksa.ch

**Keywords:** anti-inflammatory diet, arthralgia, ketogenic diet, Mediterranean diet, pain, rheumatoid arthritis, vegan diet, vegetarian diet

## Abstract

Various nutritional therapies have been proposed in rheumatoid arthritis, particularly diets rich in ω-3 fatty acids, which may lead to eicosanoid reduction. Our aim was to investigate the effect of potentially anti-inflammatory diets (Mediterranean, vegetarian, vegan, ketogenic) on pain. The primary outcome was pain on a 10 cm visual analogue scale. Secondary outcomes were C-reactive protein levels, erythrocyte sedimentation rate, health assessment questionnaire, disease activity score 28, tender/swollen joint counts, weight, and body mass index. We searched MEDLINE (OVID), Embase (Elsevier), and CINAHL for studies published from database inception to 12 November 2021. Two authors independently assessed studies for inclusion, extracted study data, and assessed the risk of bias. We performed a meta-analysis with all eligible randomized controlled trials using RevMan 5. We used mean differences or standardized mean differences and the inverse variance method of pooling using a random-effects model. The search retrieved 564 unique publications, of which we included 12 in the systematic review and 7 in the meta-analysis. All studies had a high risk of bias and the evidence was very low. The main conclusion is that anti-inflammatory diets resulted in significantly lower pain than ordinary diets (−9.22 mm; 95% CI −14.15 to −4.29; *p* = 0.0002; 7 RCTs, 326 participants).

## 1. Introduction

Rheumatoid arthritis (RA) is a chronic, autoimmune, inflammatory disorder that primarily affects the joints. Clinical manifestations of RA include joint pain, stiffness, swelling, as well as joint destructions, and systemic manifestations. RA may cause progressive joint damage and disability. Risk factors for RA are genetic and non-genetic, including smoking, changes in the microbiota, female sex, Western diet, and ethnic factors [1]. The global burden of disease study 2019 showed a global prevalence of 0.22%; 0.31% in females and 0.13% in males [2]. RA treatment comprises a multimodal approach. The pharmacologic therapy consists of disease-modifying anti-rheumatic drugs (DMARDs) and anti-inflammatory therapy with nonsteroidal anti-inflammatory drugs (NSAIDs) or glucocorticoids [1]. The non-pharmacologic measures include patient education, physiotherapy, and nutritional therapy, among others [3].

Nutritional therapy for RA aims to attenuate inflammation by altering the ratio of ω-6 to ω-3 fatty acids and increasing antioxidants. The reduction of arachidonic acid (AA), an ω-6 fatty acid, is particularly relevant. AA is the precursor of eicosanoids, which are involved in a variety of cellular functions and reactions. Eicosanoids are also mediators of inflammation, and the amount of AA released from the cell membrane determines the intensity of inflammation. When less AA is present in the cell membrane, less AA is released, and fewer eicosanoids are formed [4].

Endogenous biosynthesis produces AA and thus eicosanoids from linoleic acid adjusted to physiologic requirements. In contrast, in developed countries, AA in cell membranes mostly originates from the diet, while endogenous biosynthesis is very low, the median daily AA intake being about 210–250 mg [5]. Vegetarian diets contain less AA than diets with meat, whereas vegan diets contain virtually no AA. There is evidence from population studies that nutrients of animal origin, as consumed in high amounts in the Western diet, correlate with the occurrence of RA [6,7]. Therefore, vegetarian and vegan diets may favorably influence inflammation.

In addition, the low intake of the ω-3 fatty acid eicosapentaenoic acid (EPA) in Western societies favors the accumulation of AA. EPA lowers the AA content in cell membranes by replacing AA [8]. This results in less AA available for oxidation to inflammatory mediators. In addition, EPA is a competitive inhibitor of cyclooxygenase and lipoxygenase, two enzymes relevant to eicosanoid biosynthesis [4]. The Mediterranean diet includes weekly fish consumption but little dairy products, eggs, and red meat, thus, more fish oil (rich in ω-3 fatty acids EPA and docosahexaenoic acid (DHA)) and less AA than in the Western diet. Indeed, the role of fish oil supplements in the treatment of RA is well studied [9,10,11]. This may contribute to an anti-inflammatory effect of the Mediterranean diet.

The impact of dietary fibers on the composition and metabolic activity of the gut microbiome further contributes to the anti-inflammatory effect of vegetarian, vegan or Mediterranean diets. In RA patients, a high-fiber diet increases anti-inflammatory short-chain fatty acids, decreases pro-inflammatory cytokines, and favorably alters the gut microbiome composition [12].

The ketogenic diet may reduce eicosanoid formation through the lower generation of reactive oxygen species (ROS) of the ketone metabolism compared to the glucose metabolism [13]. ROS activate phospholipase A2 in the cell membrane of immune cells, which exclusively cleaves AA from phospholipids of the cell membrane. ROS also serve as substrates for the oxidation of AA and lead to excessive eicosanoid formation [14]. In addition, the ketogenic diet increases adenosine, which may alleviate pain and have an anti-inflammatory effect [13,15].

Our aim was to synthesize the evidence and pool the effect of the above-mentioned anti-inflammatory diets (Mediterranean, vegetarian, vegan, and ketogenic) on pain in rheumatoid arthritis in a systematic review and a meta-analysis.

## 2. Methods

We conducted this systematic review and meta-analysis according to the Preferred Reporting Items for Systematic Reviews and Meta-Analyses (PRISMA) [16].

Studies comparing the effect of a Mediterranean, vegetarian, vegan, or ketogenic diet vs. an ordinary omnivorous diet on pain in adults with RA were eligible. We included randomized and non-randomized, controlled and uncontrolled trials (including before-after studies), and observational studies (including cohort and case-control studies). We excluded reviews, conference abstracts, case reports, editorials, letters, and notes. Inclusion criteria for the studied population were adults ≥18 years of age with RA. We excluded studies on patients with non-rheumatic disorders or rheumatic disorders other than RA, and adolescents and children <18 years of age. We included studies identifying their intervention as Mediterranean, vegetarian, vegan, or ketogenic diet. We excluded non-whole diet interventions, i.e., single food items, nutrients, or supplements. The control intervention was an ordinary omnivorous diet. We included studies published in English, German, or French with no restrictions on the publication date.

We searched the electronic bibliographic databases MEDLINE via OVID, Embase via Elsevier, and CINAHL with Full Text via EBSCOhost. The last search date for all databases was 12 November 2021. In addition, we screened the reference lists of relevant publications. The search strategy included terms relating to RA-related pain and Mediterranean, vegetarian, vegan, or ketogenic diets. We searched for MeSH terms, Emtree terms, and CINAHL Subject Headings in MEDLINE, Embase, and CINAHL, respectively, and text words in title, abstract and keywords. Appendix A shows the full electronic search strategies.

Two authors independently screened titles and/or abstracts of records identified from database searches or additional sources, to identify those potentially meeting the inclusion criteria. We retrieved the full text of potentially eligible studies and two authors independently assessed them for eligibility. We resolved any disagreement over the eligibility of particular studies through discussion with a third reviewer.

We used a standardized, pilot-tested data extraction form, including information on study size, population, intervention, comparison, outcomes, study design, intervention period, and results for the main and secondary outcomes of this meta-analysis. Two authors extracted the data independently and resolved discrepancies through discussion, where necessary with a third author. We requested missing data from study authors via email. We sought baseline and endpoint data for the primary outcome pain score on a 10 cm visual analog scale (VAS) and the following secondary outcomes: C-reactive protein (CRP), erythrocyte sedimentation rate (ESR), health assessment questionnaire (HAQ) [17], disease activity score 28 (DAS28) [18,19], swollen joint count (SJC), tender joint count (TJC), weight and BMI. In addition, we sought data for the following variables: participant characteristics (number, age, sex, height), intervention and comparison characteristics, concomitant medication, and study design.

Two authors independently assessed the risk of bias in individual studies using version 2 of the Cochrane tool for assessing risk of bias in randomized trials (RoB 2) [20] and the risk of bias in non-randomized studies—of interventions (ROBINS-I) tool [21].

If possible, we summarized outcome results quantitatively in meta-analyses by using the inverse variance method based on random-effects models. We analyzed the data using RevMan 5 [22]. The principal summary measures were mean differences or standardized mean differences for outcomes measured with different instruments or on different scales (SJC and TJC). We included only randomized controlled trials (RCTs) in the meta-analysis. If the change-from-baseline SD was missing, we imputed it using a correlation coefficient from another study in the meta-analysis [23,24,25]. We assessed heterogeneity using the χ² test and the I² and τ^2^ statistic.

Since there were less than ten studies included in the meta-analysis, the risk of publication bias by evaluating the symmetry of funnel plots remained undetected [26].

Finally, we performed a transparent assessment and rating of the quality of evidence with the grading of recommendations assessment, development, and evaluation (GRADE) approach [27], using GRADEpro software [28].

## 3. Results

**Figure 1:**
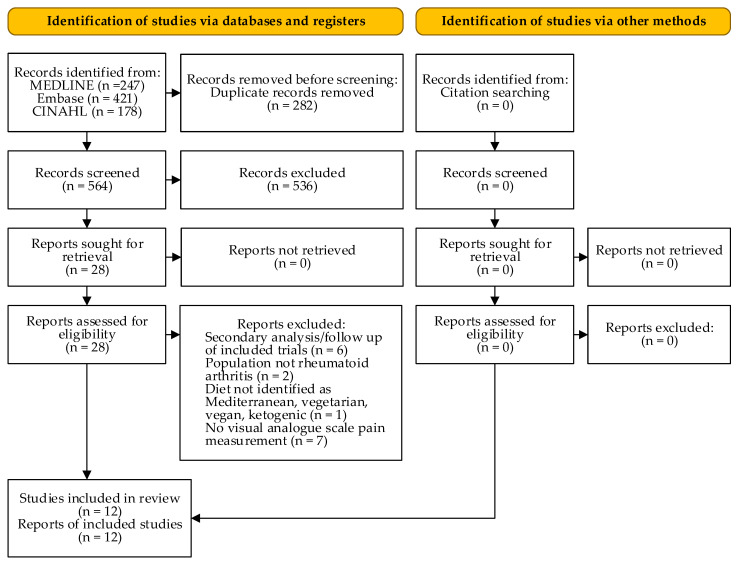
PRISMA flow diagram (according to [16]).

Figure 1 depicts the number of studies screened, assessed for eligibility, and included in the review. Table 1 shows the characteristics of the included studies. No studies assessing the effect of a ketogenic diet on pain in RA were eligible. The interventions varied in terms of included therapies (e.g., physical and drug therapy), but were usually constant over the study period.

In 1979, Sköldstam and colleagues conducted the first RCT on the effect of 7–10 days fasting followed by 9 weeks lactovegetarian diet in RA [29]. Of the 14 patients in the diet group, 8 (57%) had less pain than at baseline and planned to continue the lactovegetarian diet after the trial. However, as a group they showed no change in pain, stiffness, or use of analgesics. In 1991, Kjeldsen-Kragh and colleagues published another landmark RCT [30]. A diet group of 27 patients initially fasted (800–1260 kJ/day) for 7–10 days, followed by an individually adjusted gluten-free vegan diet for 3–5 months. Then, they gradually changed to a lactovegetarian diet for the remainder of the total study period of one year. Compared with the 26 patients in the control group, who ate ordinary mixed food, the diet group reported significant improvements in pain, duration of morning stiffness, SJC, TJC, ESR, and CRP levels, among others.

Hafström and colleagues published a RCT in 2001, in which they assessed the clinical effects of one year gluten-free vegan diet vs. non-vegan diet according to the American College of Rheumatology (ACR) response criteria (ACR20) [31]. They found a significant improvement in all clinical variables included in the ACR20 except CRP in the vegan group as compared with the non-vegan group.

In 2003, Sköldstam and colleagues conducted another RCT with 51 RA patients [32]. This time, they compared 12 weeks of Mediterranean diet with usual diet in Swedish participants. At the end of the study, patients on the Mediterranean diet reported significant decreases in pain, DAS28, HAQ score, SJC, and CRP compared to the control group. This difference was apparent only in the second half of the trial.

García-Morales and colleagues conducted the largest RCT so far [33]. RA patients were randomized in four groups: Mediterranean diet + dynamic exercise program (*n* = 26), dynamic exercise program (*n* = 37), Mediterranean diet (*n* = 40), and control (*n* = 31). The dynamic exercise program consisted of 80–90 min training sessions twice a week. After 24 weeks, the scores of physical function, vitality, mental health, bodily pain, and global health domains showed significant improvement in the dynamic exercise program group compared with the other groups.

**Figure 2:**
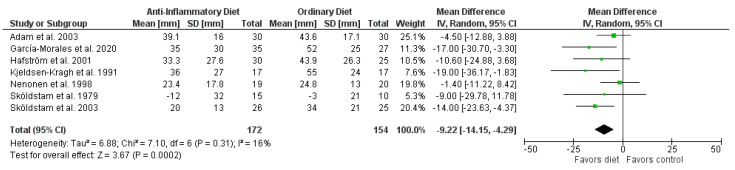
Forest plot summarizing the effect of anti-inflammatory diets on pain.

**Figure 3:**
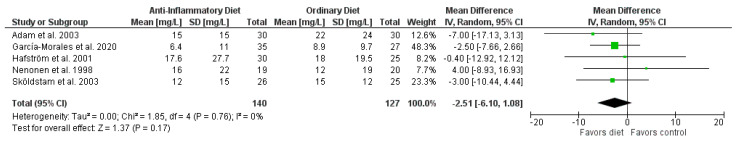
Forest plot summarizing the effect of anti-inflammatory diets on C-reactive protein levels.

The pooled results showed that overall patients on anti-inflammatory diets reported significantly less pain than patients in the control groups (mean difference (MD) −9.22 mm, 95% CI −14.15 to −4.29 mm; *p* = 0.0002; 7 RCTs, 326 participants, Figure 2), improved HAQ (−0.20 points, 95% CI −0.36 to −0.05 points; *p* = 0.01; 4 RCTs; 202 participants, Figure S2), and lower SJC (standardized mean difference (SMD) −0.60, 95% CI −1.08 to −0.11; *p* = 0.02; 4 RCTs; 214 participants, Figure S3). In addition, patients on anti-inflammatory diets lost more weight than patients in the control groups (MD −3.73 kg, 95% CI −5.45 to −2.01 kg; *p* < 0.0001; 6 RCTs; 286 participants, Figure S5) and BMI decreased (MD −1.28 kg/m^2^, 95% CI −1.89 to −0.67 kg/m^2^; *p* < 0.0001; 4 RCTs; 209 participants, Figure S6). There were no significant differences in CRP (Figure 3), ESR (Figure S1), and TJC (Figure S4). Since only two RCTs reported DAS28, we did not perform a meta-analysis for this outcome.

Subgroup analysis showed that Mediterranean diets tended to have a greater effect on pain than vegetarian or vegan diets did (−14.99 mm, 95% CI −22.87 to −7.11 mm; *p* = 0.0002; 2 RCTs, 113 participants vs. −6.13 mm, 95% CI −11.46 to−0.80 mm; *p* = 0.02; 5 RCTs, 213 participants; test for subgroup differences *p* = 0.07, Figure S7). In addition, studies with a longer intervention period tended to have greater effects (intervention period ≤3 months −6.71 mm, 95% CI −12.52 to −0.90 mm; *p* = 0.02; 4 RCTs, 175 participants vs. intervention period >3 months −15.19 mm, 95% CI −23.76 to −6.63 mm; *p* = 0.0005; 3 RCTs, 151 participants; test for subgroup differences *p* = 0.11, Figure S8).

Table 2 and Table 3 summarize the risk of bias assessment of the RCTs and non-randomized studies, respectively, for the outcome pain. All studies had a high risk of bias in the domain measurement of the outcome, since it is not possible to blind the dietary intervention and pain is a subjective, self-reported outcome. For objectively measured secondary outcomes, all RCTs had some concerns overall, since there was no information on whether the data that produced this result were analyzed in accordance with a pre-specified analysis plan. Consequently, the GRADE assessment resulted in very low or low certainty for all outcomes (Appendix B).

A search on ClinicalTrials.gov (accessed on 2 September 2021) revealed four studies by the Physicians Committee for Responsible Medicine with a vegan diet intervention in RA patients. Two of the studies are active (NCT01700881 and NCT03580681), while the others were completed in 2012 (NCT01544101) and 2018 (NCT03417648). The researchers were not able to share the unpublished results at this point, but they are summarizing and publishing the findings from several replications of the same study [45].

## 4. Discussion

Our meta-analysis showed a significant improvement in pain in RA patients on anti-inflammatory diets compared with ordinary diets. Stauffer et al. determined that for a baseline VAS of 30–49 mm, the minimal clinically important difference for improvement was 7–11 mm [46]. Therefore, the mean difference of our meta-analysis (−9.22 mm) is clinically relevant, although the 95% CI (−14.15 to −4.29 mm) might refute this. Non-randomized trials support our findings [38,39,43]. Given the level of evidence for the outcome pain, the actual effect could deviate from the estimated effect.

Subgroup analysis showed that Mediterranean diets tended to have a greater effect on pain than vegetarian or vegan diets did. However, only two RCTs intervened with a Mediterranean diet. The observational, cross-sectional study by Ingegnoli et al. found a significant negative association between Mediterranean diet adherence and pain [38]. Special consideration should be given when recommending the Mediterranean diet to RA patients, as gluten sensitivity is more common in patients with rheumatic diseases than in the general population [47], and the Mediterranean diet contains high amounts of whole grain products. None of the eligible studies investigated the effect of a ketogenic diet, which comes close to fasting in terms of metabolism, but is difficult to follow because it is restrictive in everyday life.

RCTs with significant effects tended to have a longer intervention period (13 months [30], 6 months [33], 12 weeks [32]) than RCTs with insignificant effects (3 months [36], 2–3 months [41], 9 weeks [29]). Studies investigating ω-3 fatty acids in RA found similar results [48]. This indicates that effects of dietary interventions for RA occur from three months onwards.

Intervention group patients with a higher baseline BMI [32,33] appeared to have a greater improvement in pain than patients with normal weight or borderline overweight [36,41]. However, improvement in pain did not correlate with weight loss. A previous meta-analysis concluded that obesity negatively impacts disease activity and patient-reported outcomes in RA [49].

The significant and clinically relevant improvement in the secondary outcomes HAQ and SJC confirmed the perceived improvement in pain. However, although CRP and ESR show a tendency for improvement, these results were not significant and therefore we cannot assume an underlying pathophysiological mechanism for the improved pain. Furthermore, the physician-assessed SJC improved significantly, while the TJC did not.

The effect of exercise in RA is well established [50,51]. García-Morales et al. [33] conducted a multi-arm study, including a control group without any intervention and intervention groups receiving a dynamic exercise program or Mediterranean diet only or both. There was no additional benefit of the Mediterranean diet and exercise over exercise only, suggesting that the observed effect might be the result of any lifestyle intervention vs. no intervention. Likewise, all patients in the study of Sköldstam et al. [29] participated in the usual physiotherapy and physical training on the ward and the decrease in pain was not significantly greater in the intervention group. Conversely, all participants in the study of Kjeldsen-Kragh et al. [30] were offered physiotherapy three times a week during the first four weeks of the study, yet the decrease in pain was greater in the diet group after the first month of the study. Similarly, Sköldstam et al. [32] found a significantly greater decrease in pain in the diet than the control group, although they recruited patients from the outpatient-based rehabilitation program, which includes patient education, strength and fitness training, and individual physiotherapy and occupational therapy.

The studies included mainly female patients (92%). Of note, the RA prevalence is two to three times higher in women than in men [2]. The studies included in this systematic review did not investigate differences between male and female patients. Therefore, the information is insufficient to make any conclusions.

A Cochrane review of 14 RCTs involving 837 RA patients on different diets provided little evidence of their effectiveness. However, the results of studies with different interventions and follow-up lengths were not pooled. Consequently, each study was reported individually in a separate forest plot [52]. Another meta-analysis included studies with interventions termed as low-inflammatory diet, anti-inflammatory diet, Mediterranean diet or synonyms of these in patients with osteoarthritis, RA, and seronegative arthropathies. While the physical outcome measures as well as pain scores did not favor either diet overall, the effect was significant in RA. Thus, their results were similar to the present meta-analysis in terms of patient-reported outcomes and quality of evidence, but they found a significant effect of diet on the inflammatory biomarkers interleukin-6 and CRP [3].

In the context of multimodal therapy, diets are one of many possibilities that should be offered to patients. Perception of pain varies from individual to individual and is highly subjective, and there may be a placebo effect in many patients. The influence of other factors cannot be investigated based on the current data, but it is probably high. Nevertheless, we chose perceived pain as the primary outcome of this meta-analysis because it has a positive effect on the disease burden and quality of life. Many RA patients seek adjuvant therapies to pharmacotherapy and are mainly looking for symptom and specifically pain relief [53]. Hence, the effect of nutritional therapy on pain is not only essential for patient outcome but also for compliance. We assume, however, that the effect of diet is greater with high disease activity and low medication therapy. Especially when drug therapy is exhausted, diet can be a valuable therapeutic modality with few side effects. This is particularly relevant with regard to chronic opioid use and addiction, for which RA patients are especially susceptible [54,55,56].

The limitations of this meta-analysis are in the nature of the research question, as it is not possible to blind dietary interventions and pain measured by VAS is a subjective self-reported outcome. Therefore, all studies had a high risk of bias for the primary outcome pain. In addition, publication bias was difficult to assess due to the small number of published RCTs. Apart from the two unpublished completed trials registered in ClinicalTrials.gov (accessed on 2 September 2021), there were no indications for publication bias. The risk of bias in the individual studies together with the width of the CI resulted in very low certainty of evidence in the GRADE rating.

We pooled the results of dietary interventions with Mediterranean, vegetarian, and vegan diet in this meta-analysis. Our rationale for this was that the definition of these diets differed across the studies (see Table 1). Moreover, the implementation and monitoring of diet adherence was heterogeneous. In spite of this, all studies investigated an intervention with an anti-inflammatory diet as defined in the protocol of this meta-analysis. Finally, this heterogeneity assumingly represents the actions and implementation by patients in clinical practice outside of a study setting better than strict definitions and separations of the diets. Therefore, we chose to perform a pragmatic and explorative, yet statistically more powerful meta-analysis to investigate the potential of nutritional therapy with anti-inflammatory diets. Nevertheless, we conducted a subgroup analysis on the effect of the different diet forms for the main outcome pain (Figure S7).

In conclusion, the decreased subjective pain rating of patients on anti-inflammatory diets compared with patients on ordinary diets was clinically relevant. Vegetarian, vegan, and Mediterranean diets might be beneficial for some RA patients. However, due to lack of blinding, effects on the patient-reported outcome pain might be biased.

## 5. Other Information

We registered this systematic review and meta-analysis on the international prospective register of systematic reviews (PROSPERO, registration number CRD42021223712). An additional protocol was not prepared. There were no amendments to information provided at registration.

## Figures and Tables

**Table 1:** Study characteristics.

Author Year	Population: n (% female) Age, Mean (SD/Range/IQR), y	Intervention vs. Control	Diet		Outcome, Mean (SD) Baseline; Endpoint		Study Design
					Intervention	Control	
Abendroth et al. 2010 [34]	MED: *n* = 28 (93%) Age: 60 (SD 12)	2 weeks MED vs. 7-day fasting	MED according to Leitzmann [35]: normocaloric, mostly vegetarian, whole grain diet, fruit, and vegetables 7 p/day, abundant intake of whole grain bread, pasta and rice, fish 2 p/week, exclusive use of olive and canola oil	Pain, mm CRP, mg/L HAQ DAS28 BMI, kg/m^2^	35 (27); 33 (26) 20 (27); 16 (22) 2.4 (0.8); 2.2 (0.8) 5.4 (1.4); 4.5 (1.3) 25.5 (5.8); NA	NA	non-randomized intervention study
Adam et al. 2003 [36]	AID + corn oil: *n* = 30 (93%) Age: 58 (SD 13) WD + corn oil: *n* = 30 (93%) Age: 57 (SD 13)	WD vs. AID with menhaden oil vs. corn oil crossover, 3 months each	AID: modified lactovegetarian diet, only plant-derived fats and oils, no egg yolk, dairy products with reduced fat, meat maximum 2 × 120 g/week WD: usual diet, characteristic for industrialized countries, meat, and meat products >2×/week	Pain, mm CRP, mg/L SJC, n TJC, n Weight, kg BMI, kg/m^2^	AID + corn oil 48 (21); 39 (16) 16 (15); 15 (15) 35 (4.9); 30 (4.5) 34 (5.1); 30 (4.7) 65 (11); 63 (9.3) 24.9 (0.7); 24.1 (0.7)	WD + corn oil 44 (18); 44(17) 22 (25); 22 (24) 34 (2.8); 36 (4.7) 36 (4.9); 36 (4.7) 62 (10); 63 (8.0) 23.2 (0.7); 23.3 (0.7)	RCT
García-Morales et al. 2020 [33]	MED: *n* = 40 (100%) Age: 46 (SD 13) Control: *n* = 31 (100%) Age: 49 (SD 12)	24 weeks MED + DEP vs. DEP vs. MED vs. control	MED: individualized according to Harris-Benedict BMR [37], 50% carbohydrates, 30% fats, 20% proteins, olive or canola oil as main dietary fat, whole grains (1–2 p/meal), fruits (2–4 p/day), vegetables (2–3 p/meal), fish (>2 p/week), oilseeds (1–2 p/day), legumes (>2 p/week), red meat (<2 p/week) Control: general nutritional recommendations	Pain, mm CRP, mg/L ESR, mm/h HAQ DAS28 SJC, n TJC, n Weight, kg BMI, kg/m^2^	MED: 45 (32); 35 (30) 6 (9); 6 (11) 11 (9); 11 (12) 0.5 (0.5); 0.5 (0.6) 2.2 (1.1); 2.4 (0.6) 1.0 (1.6); 0.9 (1.5) 1.4 (2.0); 2.9 (2.6) 67 (10); 64 (10) 27.2 (3.6); 26.5 (3.7)	Control: 51 (27); 52 (25) 4 (4); 9 (10) 16 (10); 18 (16) 0.9 (0.7); 0.8 (0.6) 2.6 (0.9); 2.4 (0.7) 1.4 (1.9); 2.0 (2.3) 1.5 (1.7); 0.7 (1.2) 64 (8.3); 66 (16) 27.1 (4.2); 27.6 (6.2)	RCT
Hafström et al. 2001 [31]	Vegan: *n* = 38 Age: 50 (SD 9.6) Control: *n* = 28 Age: 51 (SD 12)	1 year gluten-free vegan diet vs. non-vegan diet	Gluten-free vegan: vegetables, root vegetables, nuts and fruits, buckwheat, millet, corn, rice, and sunflower seeds. Unshelled sesame seeds in the form of sesame milk were a daily source of calcium. Non-vegan diet: variety of foods from all food groups	Pain, mm CRP, mg/L HAQ Weight, kg	46 (19); 33 (28) 23 (19); 18 (28) 1.4 (0.4); 1.1 (0.7) 66 (13); 61 (10)	46 (21); 44 (26) 25 (22); 18 (20) 1.3 (0.5); 1.2 (0.5) 68 (20); 66 (15)	RCT
Ingegnoli et al. 2020 [38]	*n* = 205 (80%) Age: Mdn 53 (IQR 44–59)	N/A (observational study on the association between the MED score and RA disease impact, activity, and comorbidities)		Pain CRP HAQ DAS28-CRP SJC TJC BMI	Univariate analysis: association between outcomes (dependent variables) and the adherence to MED (independent variable) regression coefficient (95% CI) −0.08 (−0.15, −0.01) 0.01 (−0.03, 0.05) −0.01 (−0.02, −0.001) −0.01 (−0.04, 0.01) −0.01 (−0.03, 0.01) −0.02 (−0.06, 0.02) −0.04 (−0.15,0.07)		observational, cross-sectional study
Kjeldsen-Kragh et al. 1991 [30]	Vegetarian: *n* = 27 (89%) Age: 53 (range 26–63) Control: *n* = 26 (81%) Age: 56 (range 38–78)	13 months vegetarian vs. usual diet	Vegetarian: initial 7–10 days fast (800–1260 kJ/day), afterwards reintroduction of a new food item every 2nd day, during the first 3–5 months no gluten, meat, fish, eggs, dairy products, refined sugar, citrus fruits, salt, strong spices, preservatives, alcoholic beverages, tea, coffee, afterwards reintroduction of milk, other dairy products Control: ordinary mixed food	Pain, mm HAQ Weight, kg	NA; 36 (27) NA; 1.0 (0.6) NA; 65 (11)	NA; 55 (24) NA; 1.1 (0.6) NA; 67 (11)	RCT
McDougall et al. 2002 [39]	Vegan: *n* = 24 (92%) Age: 54 (SD 11)	4 weeks vegan diet	Low-fat, vegan diet: no animal products or added fats and oils of any kind, ad libitum menus based on common starches, such as beans, breads, corn, pastas, potatoes, sweet potatoes, and rice, fresh or fresh-frozen fruits and vegetables, dehydrated cereals, soups, main entrees	Pain, mm CRP, mg/L ESR, mm/h SJC, n TJC, n Weight, kg	49 (20); 34 (20) 21 (18); 17 (17) 50 (30); 50 (28) 27 (9); 22 (8) 24 (12); 17 (16) 68 (19); 65 (18)	NA	uncontrolled, pre-post intervention study
McKellar et al. 2007 [40]	MED: *n* = 75 (100%) Age: 54 (IQR 47–64) Control: *n* = 55 (100%) Age: 53 (IQR 45–61)	6 months MED vs. healthy eating	MED: 6-week cookery course on Medi-terranean-type diet, weekly 2 h cookery class, written information on a Medi-terranean-type diet, healthy eating and recipes promoting fruits, vegetables and legumes, substitution of saturated fat with olive oil or spreads containing olive oil Control: readily available written information on healthy eating	Pain, mm CRP, mg/L ESR, mm/h HAQ DAS28 SJC, n TJC, n Weight, kg BMI, kg/m^2^	Median: 50; 50 10; 10 19; 16 1.8; 1.6 4.7; 4.4 6; 4 5; 4 66; 65 25.9; 25.4	Median: 55; 63 8.5; 8.0 19; 16 1.8; 1.9 5.0; 4.8 6; 5 6; 6 70; 73 27.7; 28.2	non-randomized intervention study
Nenonen et al. 1998 [41]	Vegan: *n* = 22 (82%) Age: 49 (SD 7) Control: *n* = 20 (95%) Age: 56 (SD 11)	2–3 months vegan vs. omnivorous diet	Vegan living food diet according to Hänninen [42]: uncooked, rich in lacto-bacilli, no animal products, no refined substances, no added salt, majority of food items soaked and sprouted (seeds and grains), fermented, bread is blended and dehydrated. Control: previous omnivorous diet	Pain, mm CRP, mg/L ESR, mm/h SJC, n TJC, n Weight, kg BMI, kg/m^2^	36 (14); 23 (18) 13 (16); 16 (22) 33 (16); 41 (22) 3.4 (2.5); 3.6 (3.0) 8.6 (4.7); 6.5 (4.7) 68 (10); 62 (9) 25.5 (4.0); 23.4 (3.5)	38 (15); 25 (13) 17 (24); 12 (19) 40 (26); 43 (26) 3.9 (3.6); 3.8 (2.8) 9.6 (4.6); 9.6 (5.2) 64 (12); 64 (11) 23.5 (3.4); 23.7 (3.5)	RCT
Sköldstam et al. 1979 [29]	Vegetarian: *n* = 16 (63%) Age: 52 (range 35–66) Control: *n* = 10 (90%) Age: 54 (range 43–65)	9 weeks vegetarian vs. normal diet	Vegetarian: initial 7–10 days fast (800 kJ/day, 3 L fruit and vegetable juices), followed by plain lactovegetarian diet, no animal or fish protein (including egg), yoghurt allowed freely, fresh milk and cream discouraged, no alcohol, tobacco, coffee, tea, restriction on salt, sugar, white flour, small quantities of grain products Control: normal diet	Pain, mm ESR, mm/h Weight, kg	35 (19), Δ-12 (32) 41 (23), Δ2.3 (11) 71 (15), Δ−2.6 (2.1)	27 (17), Δ-3 (21) 41 (20), Δ0.7 (14) 69 (9.5), Δ0.6 (2.0)	RCT
Sköldstam 1986 [43]	*n* = 20 (90%) Age: range 35–68	4 months vegan diet vs. ordinary diet	Vegan: initial 7–10 days fast, followed by diet excluding meat, fish, eggs and dairy products, refined sugar, corn flour, salt, strong spices, preservatives, alcoholic beverages, tea, coffee Control: ordinary diet	Pain, mm CRP, mg/L ESR, mm/h Weight, kg	45 (NA); 36 (NA) No change No change Δ−4.8 (0.7)	NA	uncontrolled, pre-post intervention study
Sköldstam et al. 2003 [32]	MED: *n* = 26 (81%) Age: 58 (range 33–73) Control: *n* = 25 (80%) Age: 59 (range 35–75)	12 weeks MED vs. usual diet	Cretan MED according to de Lorgeril [44]: olive and canola oil for cooking, canola-based margarine, reduced consumption of dairy products or low-fat dairy products, green or black tea Control: ordinary hospital food followed by usual diet at home.	Pain, mm CRP, mg/L ESR, mm/h HAQ DAS28 SJC, n TJC, n Weight, kg BMI, kg/m^2^	32 (20); 20 (13) 17 (20); 12 (15) 24 (15); 25 (15) 0.7 (0.5); 0.6 (0.4) 4.4 (1.2); 3.9 (1.2) 7.0 (5.6); 5.2 (5.1) 6.8 (5.9); 4.5 (5.1) 79 (14); 76 (14) 28.4 (4.9); 27.3 (4.6)	31 (20); 34 (21) 15 (14); 15 (12) 23 (15); 25 (19) 0.8 (0.6); 0.8 (0.6) 4.3 (1.4); 4.3 (1.5) 6.9 (5.0); 7.5 (5.7) 6.9 (6.3); 6.1 (6.4) 73 (13); 73 (13) 25.7 (3.6); 25.6 (3.6)	RCT

AID, anti-inflammatory diet; BMR, basal metabolic rate; CRP, C-reactive protein; DAS28, disease activity score 28 [18]; DAS28-CRP, disease activity score 28 based on C-reactive protein [19]; DEP, dynamic exercise program; ESR, erythrocyte sedimentation rate; HAQ, health assessment questionnaire [17]; IQR, interquartile range; Mdn, median; MED, Mediterranean diet; NA, not applicable or not available; p, portions; RA, rheumatoid arthritis; RCT, randomized clinical trial; SJC, swollen joint count; TJC, tender joint count; WD, Western diet; y, years.

**Table 2:** Risk of bias assessment of randomized controlled trials for the primary outcome pain.

Author Year	Randomization Process	Deviations from Interventions	Missing Outcome Data	Outcome Measurement	Selection of the Reported Result	Overall Bias
Adam et al. 2003 [36]	Some concerns	Low	Low	High	Some concerns	High
García-Morales et al. 2020 [33]	Low	Low	Low	High	Some concerns	High
Hafström et al. 2001 [31]	Some concerns	Low	Low	High	Some concerns	High
Kjeldsen-Kragh et al. 1991 [30]	Some concerns	Low	Low	High	Some concerns	High
Nenonen et al. 1998 [41]	Some concerns	Some concerns	Low	High	Some concerns	High
Sköldstam et al. 1979 [29]	Low	Some concerns	Low	High	Some concerns	High
Sköldstam et al. 2003 [32]	Low	Some concerns	Low	High	Some concerns	High

**Table 3:** Risk of bias assessment of non-randomized studies for the primary outcome pain.

Author Year	Confounding	Selection of Participants	Intervention Classification	Deviations from Interventions	Missing Outcome Data	Outcome Measurement	Selection of the Reported Result	Overall Bias
Abendroth et al. 2010 [34]	Serious	Low	Moderate	No information	No information	Serious	Moderate	Serious
Ingegnoli et al. 2020 [38]	Serious	Moderate	Low	No information	Low	Serious	Low	Serious
McDougall et al. 2002 [39]	Low	Low	Low	Low	Low	Serious	Low	Serious
McKellar et al. 2007 [40]	Low	Low	Low	No information	Low	Serious	Low	Serious
Sköldstam 1986 [43]	Low	Low	No information	Low	No information	Serious	Low	Serious

## Data Availability

The data presented in this study are available in this article and corresponding supplementary materials.

## Supplementary Materials

The following are available online at https://www.mdpi.com/article/10.3390/nu13124221/s1, Figure S1: Forest plot summarizing the effect of anti-inflammatory diets on erythrocyte sedimentation rate, Figure S2: Forest plot summarizing the effect of anti-inflammatory diets on health assessment questionnaire score, Figure S3: Forest plot summarizing the effect of anti-inflammatory diets on swollen joint count, Figure S4: Forest plot summarizing the effect of anti-inflammatory diets on tender joint count, Figure S5: Forest plot summarizing the effect of anti-inflammatory diets on weight loss, Figure S6: Forest plot summarizing the effect of anti-inflammatory diets on body mass index decrease, Figure S7: Forest plot summarizing the subgroup analysis on the effect of Mediterranean vs. vegetarian or vegan diets on pain, Figure S8: Forest plot summarizing the subgroup analysis on the effect of intervention duration on pain.

## Appendix A

### Appendix A.1. Search Strategy: MEDLINE (OVID)

((Arthritis, Rheumatoid/ or Arthritis/ or (rheuma* or arthrit* or polyarthrit*).ti,ab,kw.) and (exp Diet, Vegetarian/ or exp Vegetarians/ or exp Diet, Mediterranean/ or exp Diet, Ketogenic/ or (vegetarian* or lactovegetarian* or pescovegetarian* or pescatarian* or ovovegetarian* or ovolactovegetarian* or lactoovovegetarian* or vegan* or plant-based or (Mediterranean adj3 diet) or MedDiet or (MIND adj3 diet) or (keto* adj3 diet) or (low adj3 carb*) or (carb* adj3 restricted) or (high adj3 fat adj3 diet)).ti,ab,kw.)) not (exp Animals/ not Humans.sh)

### Appendix A.2. Search Strategy: Embase (Elsevier)

(‘rheumatoid arthritis’/de OR ‘arthritis’/de OR ‘rheumatic disease’/de OR (rheuma* OR arthrit* or polyarthrit*):ti,ab,kw) AND (‘vegetarian diet’/exp OR ‘vegetarian’/exp OR ‘Mediterranean diet’/exp OR ‘ketogenic diet’/exp OR (vegetarian* OR lactovegetarian* OR pescovegetarian* OR pescatarian* OR ovovegetarian* OR ovolactovegetarian* OR lactoovovegetarian* OR vegan* OR plant-based OR (Mediterranean NEAR/3 diet) OR MedDiet OR (MIND NEAR/3 diet) OR (keto* NEAR/3 diet) OR (low NEAR/3 carb*) OR (carb* NEAR/3 restricted) OR (high NEAR/3 fat NEAR/3 diet)):ti,ab,kw) NOT ((‘animal’/de OR ‘animal experiment’/exp OR ‘nonhuman’/de) NOT (‘human’/exp OR ‘human experiment’/de)) NOT ‘conference abstract’/it

### Appendix A.3. Search Strategy: CINAHL with Full Text (EBSCOhost)

(MH (Arthritis, Rheumatoid OR Arthritis OR Rheumatic Diseases) OR TI (rheuma* OR arthrit* OR polyarthrit*) OR AB (rheuma* OR arthrit* OR polyarthrit*)) AND (MH (Vegetarianism OR Plant-Based Diet OR Mediterranean Diet OR Diet, Ketogenic OR Diet, Low Carbohydrate) OR TI (vegetarian* OR lactovegetarian* OR pescovegetarian* OR pescatarian* OR ovovegetarian* OR ovolactovegetarian* OR lactoovovegetarian* OR vegan* OR plant-based OR (Mediterranean N3 diet) OR MedDiet OR (MIND N3 diet) OR (keto* N3 diet) OR (low N3 carb*) OR (carb* N3 restricted) OR (high N3 fat N3 diet)) OR AB (vegetarian* OR lactovegetarian* OR pescovegetarian* OR pescatarian* OR ovovegetarian* OR ovolactovegetarian* OR lactoovovegetarian* OR vegan* OR plant-based OR (Mediterranean N3 diet) OR MedDiet OR (MIND N3 diet) OR (keto* N3 diet) OR (low N3 carb*) OR (carb* N3 restricted) OR (high N3 fat N3 diet))).

## Appendix B

**Table A1:** Grading of Recommendations Assessment, Development and Evaluation (GRADE) rating.

Outcome	Certainty Assessment							No. of Patients		Absolute Effect (95% CI)	Certainty
	No. of Studies	Study Design	Risk of Bias	Inconsistency	Indirectness	Imprecision	Other Considerations	Anti-Inflam- matory Diet	Ordinary Diet		
Pain	7	randomized trials	very serious ^a^	not serious	not serious	serious ^b^	none	172	154	MD 9.22 lower (14.15 lower to 4.29 lower)	⊕◯◯◯ very low
CRP	5	randomized trials	serious ^c^	not serious	not serious	very serious ^d^	none	140	127	MD 2.51 lower (6.10 lower to 1.08 higher)	⊕◯◯◯ very low
ESR	4	randomized trials	serious ^e^	not serious	not serious	serious ^f^	none	95	82	MD 2.9 lower (7.67 lower to 1.87 higher)	⊕⊕◯◯ low
HAQ	4	randomized trials	very serious ^g^	not serious	not serious	serious ^f^	none	108	94	MD 0.20 lower (0.36 lower to 0.05 lower)	⊕⊕◯◯ low
SJC	4	randomized trials	very serious ^h^	serious ^i^	not serious	not serious	none	112	102	SMD 0.6 lower (1.08 lower to 0.11 lower)	⊕◯◯◯ very low
TJC	4	randomized trials	very serious ^h^	very serious ^j^	not serious	serious ^f^	none	110	102	SMD 0.39 lower (1.17 lower to 0.39 higher)	⊕◯◯◯ very low
Weight loss	6	randomized trials	serious ^k^	very serious ^j^	not serious	not serious	none	152	134	MD 3.73 lower (5.45 lower to 2.01 lower)	⊕◯◯◯ very low
BMI decrease	4	randomized trials	serious ^l^	very serious ^j^	not serious	not serious	none	93	99	MD 1.28 lower (1.89 lower to 0.67 lower)	⊕◯◯◯ very low

CRP, C-reactive protein; ESR, erythrocyte sedimentation rate; HAQ, health assessment questionnaire; MD, mean difference; SJC, swollen joint count; SMD, standardized mean difference; TJC, tender joint count. ^a^ All 7 trials had high risk of bias, overall. Some concerns were noted regarding the randomization process in 4 trials. Some concerns were noted due to deviations from the intended intervention in 3 trials. In all 7 trials a high risk of bias was noted regarding the outcome measurement. ^b^ Imprecision was downgraded by 1 level because the 95% confidence interval of the mean difference was sufficiently wide that the estimate could also refute the effectiveness of the intervention assuming a clinically important difference of 7–11 units on the VAS. ^c^ All 5 trials had some concerns regarding the overall bias. Some concerns were noted regarding the randomization process in 3 trials. Some concerns were noted due to deviations from the intended intervention in 2 trials. ^d^ Imprecision was downgraded by 2 levels because the 95% confidence interval of the mean difference was sufficiently wide that the estimate could also refute the effectiveness of the intervention and the sample size of the meta-analysis was too small. ^e^ All 4 trials had some concerns regarding the overall bias. Some concerns were noted regarding the randomization process in 1 trial. Some concerns were noted due to deviations from the intended intervention in 3 trials. ^f^ Imprecision was downgraded by 1 level because the 95% confidence interval of the mean difference was sufficiently wide that the estimate could also refute the effectiveness of the intervention. ^g^ All 4 trials had high risk of bias overall. Some concerns were noted regarding the randomization process in 2 trials. Some concerns were noted due to deviations from the intended intervention in 1 trial. In all 4 trials high risk of bias was noted regarding the outcome measurement. ^h^ All 4 trials had high risk of bias overall. Some concerns were noted regarding the randomization process in 2 trials. Some concerns were noted due to deviations from the intended intervention in 2 trial. In all 4 trials a high risk of bias was noted regarding the outcome measurement. ^i^ Inconsistency was downgraded by 1 level because the I² statistic may represent substantial heterogeneity. ^j^ Inconsistency was downgraded by 2 levels because the I² statistic may represent substantial heterogeneity and confidence intervals for the results of individual studies have poor overlap. ^k^ All 6 trials had some concerns regarding overall bias. Some concerns were noted regarding the randomization process in 3 trials. Some concerns were noted due to deviations from the intended intervention in 3 trials. ^l^ All 4 trials had some concerns regarding the overall bias. Some concerns were noted regarding the randomization process in 2 trials. Some concerns were noted due to deviations from the intended intervention in 2 trials.
