# Supplementary material for: Effect of Anti-Inflammatory Diets on Pain in Rheumatoid Arthritis: A Systematic Review and Meta-Analysis
Source: Nutrients. 2021 Nov 24;13(12):4221. doi: 10.3390/nu13124221 (PMC8706441; doi:10.3390/nu13124221)
Supplement: Supplementary file 1 [file nutrients-13-04221-s001.zip › nutrients-1418065-supplementary.pdf]

Supplementary Materials

Systematic Review

Effect of Anti-Inflammatory Diets on Pain in Rheumatoid Arthritis: A Systematic Review and Meta-Analysis

Katja A. Schönenberger [1,2,*], Anne-Catherine Schüpfer [1], Viktoria L. Gloy [3], Paul Hasler [4], Zeno Stanga [1], Nina Kaegi-Braun [5], Emilie Reber [1]

1 Department of Diabetes, Endocrinology, Nutritional Medicine and Metabolism, Inselspital, Bern University Hospital, University of Bern, 3010 Bern, Switzerland; anne-catherine.schuepfer@students.unibe.ch (A.-C.S.); zeno.stanga@insel.ch (Z.S.); emilie.reber@insel.ch (E.R.)
2 Division of Clinical Pharmacy and Epidemiology, Department of Pharmaceutical Sciences, University of Basel, 4031 Basel, Switzerland
3 Basel Institute for Clinical Epidemiology and Biostatistics, Department of Clinical Research, University Hospital Basel, University of Basel, 4031 Basel, Switzerland; viktoria.gloy@usb.ch
4 Division of Rheumatology, Medical University Department, University of Basel Medical Faculty, Kantonsspital Aarau, 5001 Aarau, Switzerland; paul.hasler@ksa.ch
5 Division of General Internal and Emergency Medicine, Medical University Department, University of Basel Medical Faculty, Kantonsspital Aarau, 5001 Aarau, Switzerland; nina.kaegi@ksa.ch
* Correspondence: katja.schoenenberger@extern.insel.ch; Tel.: +41-31-664-21-05

Study or Subgroup	Anti-Inflammatory Diet Mean [mm/h]	SD [mm/h]	Total	Ordinary Diet Mean [mm/h]	SD [mm/h]	Total	Weight	Mean Difference IV, Random, 95% CI
García-Morales et al. 2020	11.1	11.7	35	18	16.1	27	43.9%	-6.90 [-14.10, 0.30]
Nenonen et al. 1998	41	22	19	43	26	20	10.0%	-2.00 [-17.09, 13.09]
Sköldstam et al. 1979	2.3	11.1	15	0.7	14.4	10	20.5%	1.60 [-8.95, 12.15]
Sköldstam et al. 2003	25	15	26	25	19	25	25.7%	0.00 [-9.42, 9.42]
**Total (95% CI)**			**95**			**82**	**100.0%**	**-2.90 [-7.67, 1.87]**

Heterogeneity: Tau² = 0.00; Chi² = 2.26, df = 3 (P = 0.52); I² = 0%
Test for overall effect: Z = 1.19 (P = 0.23)

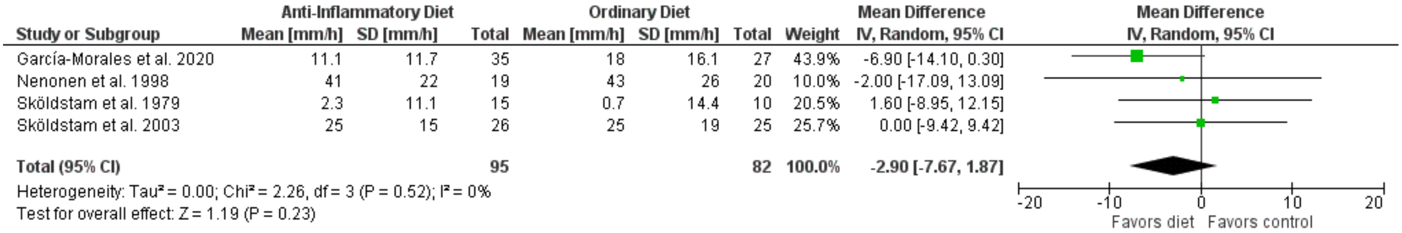

Figure S1. Forest plot summarizing the effect of anti-inflammatory diets on erythrocyte sedimentation rate

Study or Subgroup	Anti-Inflammatory Diet Mean	SD	Total	Ordinary Diet Mean	SD	Total	Weight	Mean Difference IV, Random, 95% CI
García-Morales et al. 2020	0.5	0.6	35	0.8	0.6	27	27.3%	-0.30 [-0.60, 0.00]
Hafström et al. 2001	1.1	0.7	30	1.2	0.53	25	23.4%	-0.10 [-0.43, 0.23]
Kjeldsen-Kragh et al. 1991	1	0.6	17	1.2	0.5	17	18.0%	-0.20 [-0.57, 0.17]
Sköldstam et al. 2003	0.6	0.4	26	0.8	0.6	25	31.4%	-0.20 [-0.48, 0.08]
**Total (95% CI)**			**108**			**94**	**100.0%**	**-0.20 [-0.36, -0.05]**

Heterogeneity: Tau² = 0.00; Chi² = 0.78, df = 3 (P = 0.85); I² = 0%
Test for overall effect: Z = 2.54 (P = 0.01)

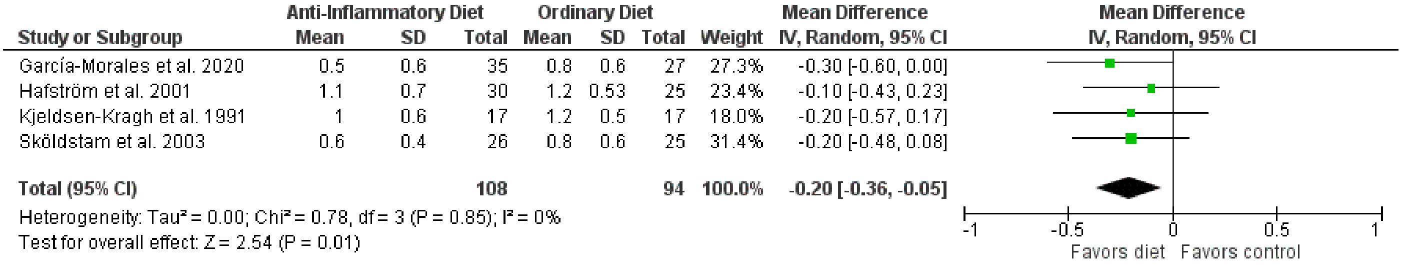

Figure S2. Forest plot summarizing the effect of anti-inflammatory diets on health assessment questionnaire score

Study or Subgroup	Anti-Inflammatory Diet Mean	SD	Total	Ordinary Diet Mean	SD	Total	Weight	Std. Mean Difference IV, Random, 95% CI
Adam et al. 2003	30	4.5	30	36	4.7	30	25.1%	-1.29 [-1.85, -0.73]
García-Morales et al. 2020	0.9	1.5	37	2	2.3	27	26.7%	-0.58 [-1.09, -0.07]
Nenonen et al. 1998	3.6	3	19	3.8	2.8	20	23.1%	-0.07 [-0.70, 0.56]
Sköldstam et al. 2003	5.2	5.1	26	7.5	5.7	25	25.2%	-0.42 [-0.97, 0.14]
**Total (95% CI)**			**112**			**102**	**100.0%**	**-0.60 [-1.08, -0.11]**

Heterogeneity: Tau² = 0.16; Chi² = 8.96, df = 3 (P = 0.03); I² = 67%
Test for overall effect: Z = 2.42 (P = 0.02)

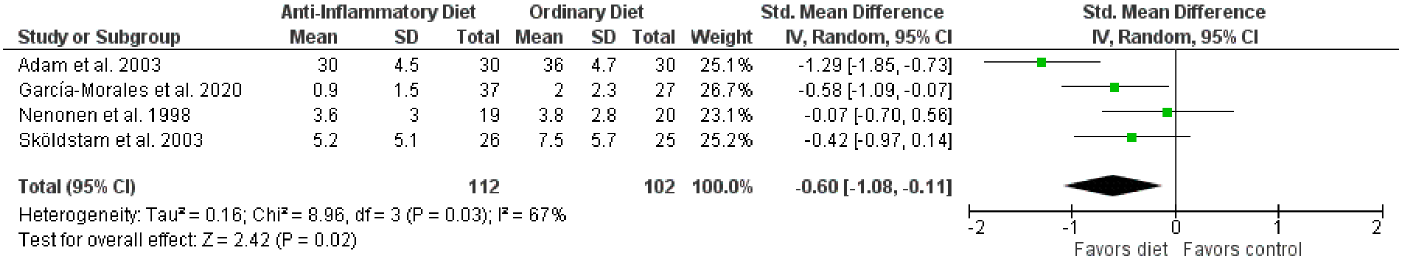

Figure S3. Forest plot summarizing the effect of anti-inflammatory diets on swollen joint count

Study or Subgroup	Anti-Inflammatory Diet Mean	SD	Total	Ordinary Diet Mean	SD	Total	Weight	Std. Mean Difference IV, Random, 95% CI
Adam et al. 2003	30	4.7	30	36	4.7	30	25.1%	-1.26 [-1.82, -0.70]
García-Morales et al. 2020	2.9	2.6	35	1.7	1.2	27	25.6%	0.56 [0.05, 1.07]
Nenonen et al. 1998	6.5	4.7	19	9.6	5.2	20	24.1%	-0.61 [-1.26, 0.03]
Sköldstam et al. 2003	4.5	5.1	26	6.1	6.4	25	25.2%	-0.27 [-0.82, 0.28]
**Total (95% CI)**			**110**			**102**	**100.0%**	**-0.39 [-1.17, 0.39]**

Heterogeneity: Tau² = 0.55; Chi² = 23.11, df = 3 (P < 0.0001); I² = 87%
Test for overall effect: Z = 0.97 (P = 0.33)

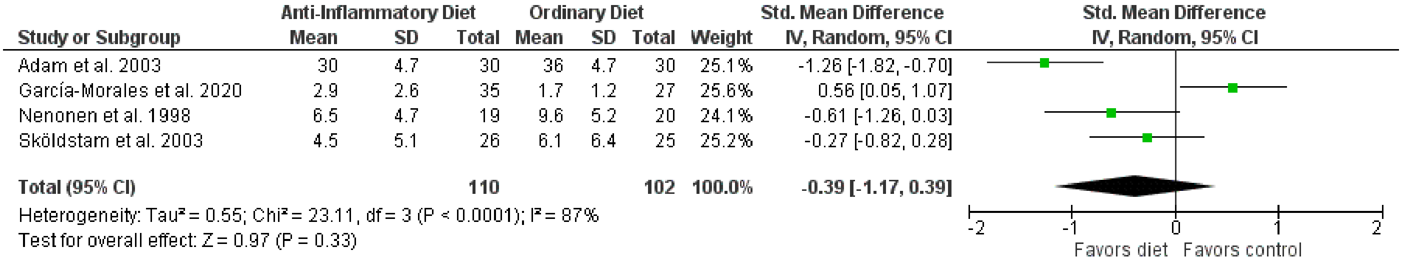

Figure S4. Forest plot summarizing the effect of anti-inflammatory diets on tender joint count

Study or Subgroup	Anti-Inflammatory Diet Mean [kg]	SD [kg]	Total	Ordinary Diet Mean [kg]	SD [kg]	Total	Weight	Mean Difference IV, Random, 95% CI
Adam et al. 2003	-2.1	2.2	30	0.4	2.2	30	18.4%	-2.50 [-3.61, -1.39]
García-Morales et al. 2020	-2.4	1.8	35	1.7	7.4	27	12.9%	-4.10 [-6.95, -1.25]
Hafström et al. 2001	-4.5	3	28	-1.4	5	25	14.9%	-3.10 [-5.35, -0.85]
Nenonen et al. 1998	-6.2	2	19	0.6	1.2	19	18.6%	-6.80 [-7.85, -5.75]
Sköldstam et al. 1979	-2.6	2.1	15	0.6	2	10	16.9%	-3.20 [-4.83, -1.57]
Sköldstam et al. 2003	-3	2.6	25	-0.4	1.3	23	18.3%	-2.60 [-3.75, -1.45]
**Total (95% CI)**			**152**			**134**	**100.0%**	**-3.73 [-5.45, -2.01]**

Heterogeneity: Tau² = 3.87; Chi² = 41.55, df = 5 (P < 0.00001); I² = 88%
Test for overall effect: Z = 4.25 (P < 0.0001)

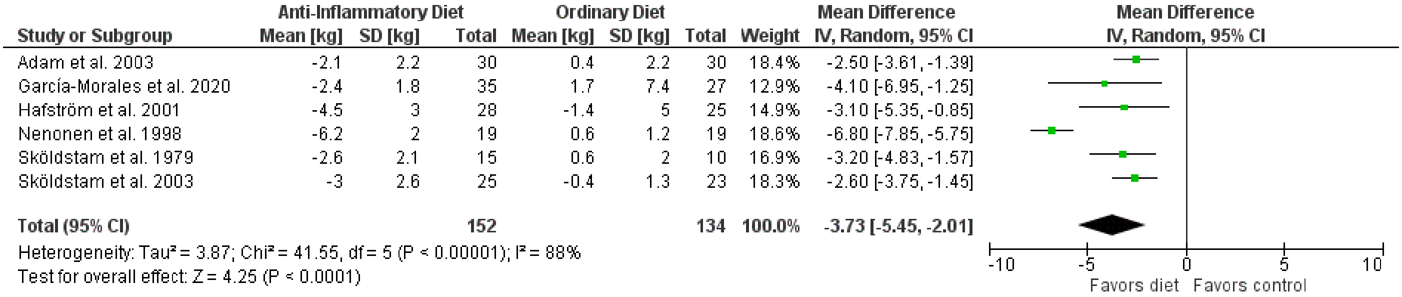

Figure S5. Forest plot summarizing the effect of anti-inflammatory diets on weight loss
SD for Adam et al. 2003, García-Morales et al. 2020, Hafström et al. 2001, and Sköldstam et al. 2003 imputed from Nenonen et al. 1998 [1-3].

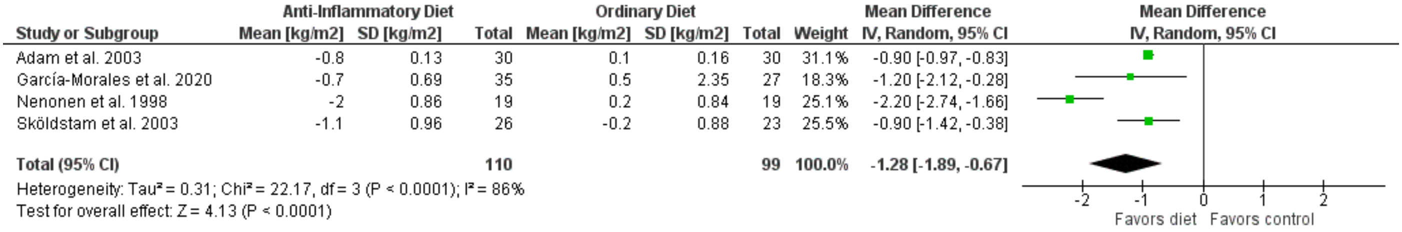

Figure S6. Forest plot summarizing the effect of anti-inflammatory diets on body mass index decrease SD for Adam et al. 2003, García-Morales et al. 2020, and Sköldstam et al. 2003 imputed from Nenonen et al. 1998 [1-3].

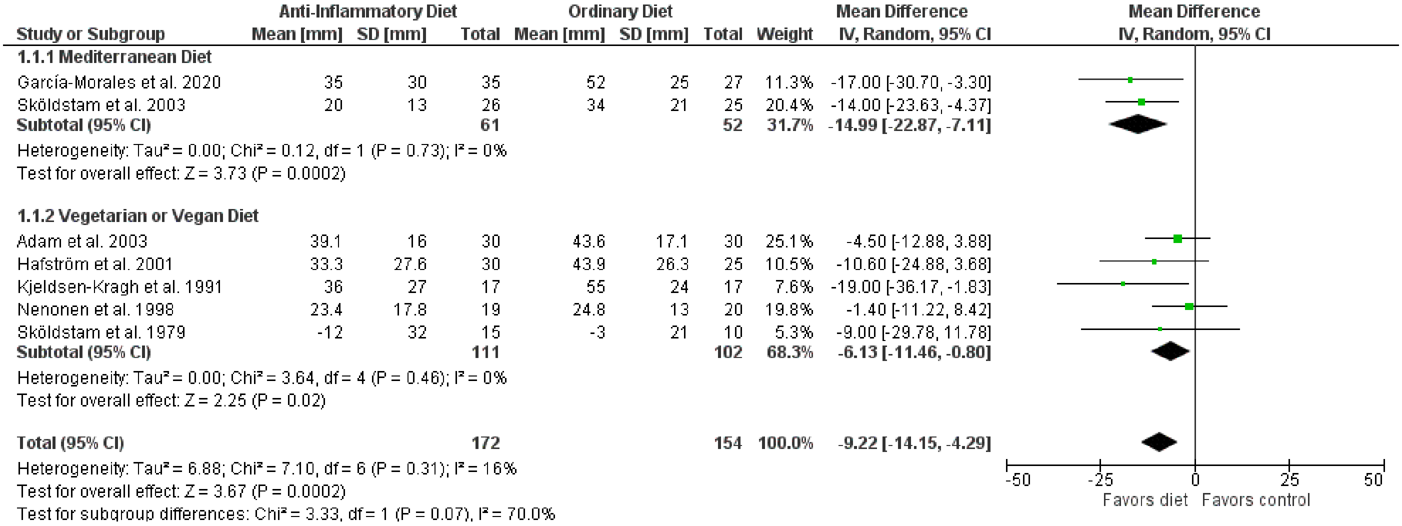

Figure S7. Forest plot summarizing the subgroup analysis on the effect of Mediterranean vs. vegetarian or vegan diets on pain

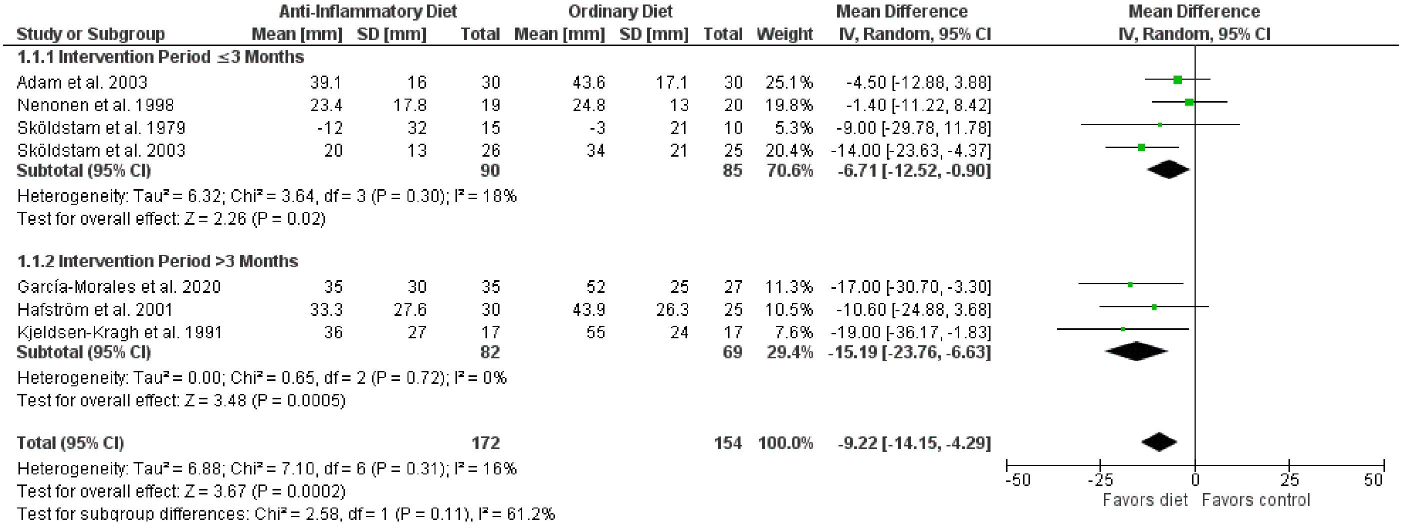

Figure S8. Forest plot summarizing the subgroup analysis on the effect of intervention duration on pain

References

1. Follmann, D.; Elliott, P.; Suh, I.; Cutler, J. Variance imputation for overviews of clinical trials with continuous response. *J Clin Epidemiol* **1992**, *45*, 769-773, doi:10.1016/0895-4356(92)90054-q.
2. Abrams, K.R.; Gillies, C.L.; Lambert, P.C. Meta-analysis of heterogeneously reported trials assessing change from baseline. *Stat Med* **2005**, *24*, 3823-3844, doi:10.1002/sim.2423.
3. Chapter 6: Choosing effect measures and computing estimates of effect. Higgins, J.P.T.; Li, T.; Deeks, J.J.; Eds. In: *Cochrane Handbook for Systematic Reviews of Interventions* version 6.2 (updated February 2021). Higgins, J.P.T.; Thomas, J.; Chandler, J.; Cumpston, M.; Li, T.; Page, M.J.; Welch, V.A.; Eds. Cochrane, 2021. Available from www.training.cochrane.org/handbook.
